# Supplementary material for: A comprehensive database for integrated analysis of omics data in autoimmune diseases
Source: BMC Bioinformatics. 2021 Jun 24;22:343. doi: 10.1186/s12859-021-04268-4 (PMC8223391; doi:10.1186/s12859-021-04268-4)
Supplement: Supplementary file 1 — Additional file 1. Description of the datasets included in the ADEx database. This table contains information about each study included in ADEx, with disease, platform, sample size and reference (if available). [file 12859_2021_4268_MOESM1_ESM.pdf]

Additional file 1. Description of the datasets included in the ADEx database. This table contains information about each study included in ADEx, with disease, platform, sample size and reference (if available).

Dataset	Studied disease	Experimental strategy	Platform	Sample size	Reference
GSE10325	SLE	Expression profiling by array	[HG-U133A] Affymetrix Human Genome U133A Array	67	[1]
GSE104174	SSc	Expression profiling by high throughput sequencing	Illumina HiSeq 2500 (Homo sapiens)	72	[2]
GSE108497	SLE	Expression profiling by array	Illumina HumanHT-12 V4.0 expression beadchip	512	NA
GSE110007	SjS	Methylation profiling by array	Illumina HumanMethylation450 BeadChip (HumanMethylation450_15017482)	31	[3]
GSE110169	SLE, RA	Expression profiling by array	[HG-U219] Affymetrix Human Genome U219 Array	234	[4]
GSE110174	SLE	Expression profiling by array	[HT_HG-U133_Plus_PM] Affymetrix HT HG-U133+ PM Array Plate	154	[4]
GSE110607	SLE	Methylation profiling by genome tiling array	Illumina HumanMethylation450 BeadChip (HumanMethylation450_15017482)	104	[5]
GSE110914	T1D	Expression profiling by high throughput sequencing	Illumina HiSeq 2500 (Homo sapiens)	42	[6]
GSE112341	T1D	Expression profiling by high throughput sequencing	Illumina HiSeq 2500 (Homo sapiens)	22	[7]
GSE117931	SSc	Expression profiling by array, Methylation profiling by genome tiling array	llumina HumanHT-12 WG-DASL V4.0 R2 expression beadchip, Illumina HumanMethylation450 BeadChip (HumanMethylation450_15017482)	74	NA

Dataset	Studied disease	Experimental strategy	Platform	Sample size	Reference
GSE11907	SLE	Expression profiling by array	[HG-U133A] Affymetrix Human Genome U133A Array [HG-U133B] Affymetrix Human Genome U133B Array	546	[8]
GSE12021	RA	Expression profiling by array	[HG-U133A] Affymetrix Human Genome U133A Array [HG-U133B] Affymetrix Human Genome U133B Array	57	[9]
GSE124073	SSc	Expression profiling by high throughput sequencing	Illumina HiSeq 2000 (Homo sapiens)	73	[10]
GSE124939	SLE	Expression profiling by high throughput sequencing	Illumina HiSeq 4000 (Homo sapiens)	72	[11]
GSE13887	SLE	Expression profiling by array	[HG-U133_Plus_2] Affymetrix Human Genome U133 Plus 2.0 Array	27	[12]
GSE23117	SjS	Expression profiling by array	[HG-U133_Plus_2] Affymetrix Human Genome U133 Plus 2.0 Array	15	[13]
GSE24706	SLE	Expression profiling by array	Illumina HumanWG-6 v3.0 expression beadchip	48	[14]
GSE27895	SLE	Methylation profiling by array	Illumina HumanMethylation27 BeadChip (HumanMethylation27_270596_v.1.2)	23	[15]
GSE30153	SLE	Expression profiling by array	[HG-U133_Plus_2] Affymetrix Human Genome U133 Plus 2.0 Array	26	[16]
GSE38351	SLE,RA	Expression profiling by array	[HG-U133A] Affymetrix Human Genome U133A Array [HG-U133_Plus_2] Affymetrix Human Genome U133 Plus 2.0 Array	74	[17]
GSE40611	SjS	Expression profiling by array	[HG-U133_Plus_2] Affymetrix Human Genome U133 Plus 2.0 Array	49	[18]

Dataset	Studied disease	Experimental strategy	Platform	Sample size	Reference
GSE42861	RA	Methylation profiling by array	Illumina HumanMethylation450 BeadChip (HumanMethylation450_15017482)	689	[19]
GSE45291	SLE,RA	Expression profiling by array	[HT_HG-U133_Plus_PM] Affymetrix HT HG-U133+ PM Array Plate	805	[20]
GSE50772	SLE	Expression profiling by array	[HG-U133_Plus_2] Affymetrix Human Genome U133 Plus 2.0 Array	81	[21]
GSE51092	SjS	Expression profiling by array	Illumina HumanWG-6 v3.0 expression beadchip	222	[22]
GSE55098	T1D	Expression profiling by array	[HG-U133_Plus_2] Affymetrix Human Genome U133 Plus 2.0 Array	22	[23]
GSE55235	RA	Expression profiling by array	[HG-U133A] Affymetrix Human Genome U133A Array	30	[24]
GSE55457	RA	Expression profiling by array	[HG-U133A] Affymetrix Human Genome U133A Array	33	[24]
GSE56606	T1D	Methylation profiling by array	Illumina HumanMethylation27 BeadChip (HumanMethylation27_270596_v.1.2)	100	[25]
GSE56649	RA	Expression profiling by array	[HG-U133_Plus_2] Affymetrix Human Genome U133 Plus 2.0 Array	22	[26]
GSE57383	RA	Expression profiling by array	[HT_HG-U133_Plus_PM] Affymetrix HT HG-U133+ PM Array Plate	112	[27]
GSE57869	SLE	Methylation profiling by array	Illumina HumanMethylation27 BeadChip (HumanMethylation27_270596_v.1.2)	12	[28]
GSE59250	SLE	Methylation profiling by array	Illumina HumanMethylation450 BeadChip (HumanMethylation450_15017482)	434	[29]

Dataset	Studied disease	Experimental strategy	Platform	Sample size	Reference
GSE60424	T1D	Expression profiling by high throughput sequencing	Illumina HiScanSQ (Homo sapiens)	134	[30]
GSE61635	SLE	Expression profiling by array	[HG-U133_Plus_2] Affymetrix Human Genome U133 Plus 2.0 Array	129	NA
GSE63903	SSc	Expression profiling by array	Illumina HumanHT-12 V4.0 expression beadchip	14	[31]
GSE65010	RA	Expression profiling by array	[HG-U133_Plus_2] Affymetrix Human Genome U133 Plus 2.0 Array	48	[32]
GSE65391	SLE	Expression profiling by array	Illumina HumanHT-12 V4.0 expression beadchip	996	[33]
GSE71841	RA	Methylation profiling by array	Illumina HumanMethylation450 BeadChip (HumanMethylation450_15017482)	24	NA
GSE72509	SLE	Expression profiling by high throughput sequencing	Illumina HiSeq 2500 (Homo sapiens)	117	[34]
GSE7451	SjS	Expression profiling by array	[HG-U133_Plus_2] Affymetrix Human Genome U133 Plus 2.0 Array	20	[35]
GSE77298	RA	Expression profiling by array	[HG-U133_Plus_2] Affymetrix Human Genome U133 Plus 2.0 Array	23	[36]
GSE80183	SLE	Expression profiling by high throughput sequencing	Illumina HiSeq 2000 (Homo sapiens)	16	[37]
GSE82221	SLE	Expression profiling by array, Methylation profiling by genome tiling array	Illumina HumanHT-12 V4.0 expression beadchip, Illumina HumanMethylation450 BeadChip (HumanMethylation450_15017482)	110	[38]
GSE84844	SjS	Expression profiling by array	[HG-U133_Plus_2] Affymetrix Human Genome U133 Plus 2.0 Array	60	[39]

Dataset	Studied disease	Experimental strategy	Platform	Sample size	Reference
GSE87095	RA	Methylation profiling by array	Illumina HumanMethylation450 BeadChip (HumanMethylation450_15017482)	122	[40]
GSE89408	RA	Expression profiling by high throughput sequencing	Illumina HiSeq 2000 (Homo sapiens)	218	[41]
GSE90081	RA	Expression profiling by high throughput sequencing	Illumina HiSeq 2000 (Homo sapiens)	24	[42]
GSE93683	SjS	Expression profiling by array	[HG-U133_Plus_2] Affymetrix Human Genome U133 Plus 2.0 Array	48	[39]
GSE95065	SSc	Expression profiling by array	[HG-U133A_2] Affymetrix Human Genome U133A 2.0 Array (HGU133A2 Hs ENTREZG 19.0.0)	33	NA

References

1. Hutcheson J, Scatizzi JC, Siddiqui AM, Haines GK, Wu T, Li Q-Z, et al. Combined deficiency of proapoptotic regulators Bim and Fas results in the early onset of systemic autoimmunity. Immunity. 2008;28:206–17.

2. Moreno-Moral A, Bagnati M, Koturan S, Ko J-H, Fonseca C, Harmston N, et al. Changes in macrophage transcriptome associate with systemic sclerosis and mediate GSDMA contribution to disease risk. Ann Rheum Dis. 2018;77:596–601.

3. Cole MB, Quach H, Quach D, Baker A, Taylor KE, Barcellos LF, et al. Epigenetic Signatures of Salivary Gland Inflammation in Sjögren’s Syndrome. Arthritis Rheumatol Hoboken NJ. 2016;68:2936–44.

4. Hu Y, Carman JA, Holloway D, Kansal S, Fan L, Goldstine C, et al. Development of a Molecular Signature to Monitor Pharmacodynamic Responses Mediated by In Vivo Administration of Glucocorticoids. Arthritis Rheumatol Hoboken NJ. 2018;70:1331–42.

5. Ulff-Møller CJ, Asmar F, Liu Y, Svendsen AJ, Busato F, Grønbaek K, et al. Twin DNA Methylation Profiling Reveals Flare-Dependent Interferon Signature and B Cell Promoter Hypermethylation in Systemic Lupus Erythematosus. Arthritis Rheumatol Hoboken NJ. 2018;70:878–90.

6. Vecchio F, Lo Buono N, Stabilini A, Nigi L, Dufort MJ, Geyer S, et al. Abnormal neutrophil signature in the blood and pancreas of presymptomatic and symptomatic type 1 diabetes. JCI Insight. 2018;3.

7. Gao P, Uzun Y, He B, Salamati SE, Coffey JKM, Tsalikian E, et al. Risk variants disrupting enhancers of TH1 and TREG cells in type 1 diabetes. Proc Natl Acad Sci U S A. 2019;116:7581–90.

8. Chaussabel D, Quinn C, Shen J, Patel P, Glaser C, Baldwin N, et al. A modular analysis framework for blood genomics studies: application to systemic lupus erythematosus. Immunity. 2008;29:150–64.

9. Huber R, Hummert C, Gausmann U, Pohlers D, Koczan D, Guthke R, et al. Identification of intra-group, inter-individual, and gene-specific variances in mRNA expression profiles in the rheumatoid arthritis synovial membrane. Arthritis Res Ther. 2008;10:R98.

10. Mariotti B, Servaas NH, Rossato M, Tamassia N, Cassatella MA, Cossu M, et al. The Long Non-coding RNA NRIR Drives IFN-Response in Monocytes: Implication for Systemic Sclerosis. Front Immunol. 2019;10:100.

11. Tsoi LC, Hile GA, Berthier CC, Sarkar MK, Reed TJ, Liu J, et al. Hypersensitive IFN Responses in Lupus Keratinocytes Reveal Key Mechanistic Determinants in Cutaneous Lupus. J Immunol Baltim Md 1950. 2019;202:2121–30.

12. Fernandez DR, Telarico T, Bonilla E, Li Q, Banerjee S, Middleton FA, et al. Activation of mammalian target of rapamycin controls the loss of TCRzeta in lupus T cells through HRES-1/Rab4-regulated lysosomal degradation. J Immunol Baltim Md 1950. 2009;182:2063–73.

13. Greenwell-Wild T, Moutsopoulos NM, Gliozzi M, Kapsogeorgou E, Rangel Z, Munson PJ, et al. Chitinases in the salivary glands and circulation of patients with Sjögren's syndrome: macrophage harbingers of disease severity. Arthritis Rheum. 2011;63:3103–15.

14. Li Q-Z, Karp DR, Quan J, Branch VK, Zhou J, Lian Y, et al. Risk factors for ANA positivity in healthy persons. Arthritis Res Ther. 2011;13:R38.

15. Jeffries MA, Dozmorov M, Tang Y, Merrill JT, Wren JD, Sawalha AH. Genome-wide DNA methylation patterns in CD4+ T cells from patients with systemic lupus erythematosus. Epigenetics. 2011;6:593–601.

16. Garaud J-C, Schickel J-N, Blaison G, Knapp A-M, Dembele D, Ruer-Laventie J, et al. B cell signature during inactive systemic lupus is heterogeneous: toward a biological dissection of lupus. PloS One. 2011;6:e23900.

17. Smiljanovic B, Grün JR, Biesen R, Schulte-Wrede U, Baumgrass R, Stuhlmüller B, et al. The multifaceted balance of TNF-α and type I/II interferon responses in SLE and RA: how monocytes manage the impact of cytokines. J Mol Med Berl Ger. 2012;90:1295–309.

18. Horvath S, Nazmul-Hossain ANM, Pollard RPE, Kroese FGM, Vissink A, Kallenberg CGM, et al. Systems analysis of primary Sjögren's syndrome pathogenesis in salivary glands identifies shared pathways in human and a mouse model. Arthritis Res Ther. 2012;14:R238.

19. Liu Y, Aryee MJ, Padyukov L, Fallin MD, Hesselberg E, Runarsson A, et al. Epigenome-wide association data implicate DNA methylation as an intermediary of genetic risk in rheumatoid arthritis. Nat Biotechnol. 2013;31:142–7.

20. Bienkowska J, Allaire N, Thai A, Goyal J, Plavina T, Nirula A, et al. Lymphotoxin-LIGHT pathway regulates the interferon signature in rheumatoid arthritis. PloS One. 2014;9:e112545.

21. Kennedy WP, Maciuca R, Wolslegel K, Tew W, Abbas AR, Chaivorapol C, et al. Association of the interferon signature metric with serological disease manifestations but not global activity scores in multiple cohorts of patients with SLE. Lupus Sci Med. 2015;2:e000080.

22. Lessard CJ, Li H, Adrianto I, Ice JA, Rasmussen A, Grundahl KM, et al. Variants at multiple loci implicated in both innate and adaptive immune responses are associated with Sjögren's syndrome. Nat Genet. 2013;45:1284–92.

23. Yang M, Ye L, Wang B, Gao J, Liu R, Hong J, et al. Decreased miR-146 expression in peripheral blood mononuclear cells is correlated with ongoing islet autoimmunity in type 1 diabetes patients 1miR-146. J Diabetes. 2015;7:158–65.

24. Woetzel D, Huber R, Kupfer P, Pohlers D, Pfaff M, Driesch D, et al. Identification of rheumatoid arthritis and osteoarthritis patients by transcriptome-based rule set generation. Arthritis Res Ther. 2014;16:R84.

25. Rakyan VK, Beyan H, Down TA, Hawa MI, Maslau S, Aden D, et al. Identification of type 1 diabetes-associated DNA methylation variable positions that precede disease diagnosis. PLoS Genet. 2011;7:e1002300.

26. Ye H, Zhang J, Wang J, Gao Y, Du Y, Li C, et al. CD4 T-cell transcriptome analysis reveals aberrant regulation of STAT3 and Wnt signaling pathways in rheumatoid arthritis: evidence from a case-control study. Arthritis Res Ther. 2015;17:76.

27. Rosenberg A, Fan H, Chiu YG, Bolce R, Tabechian D, Barrett R, et al. Divergent gene activation in peripheral blood and tissues of patients with rheumatoid arthritis, psoriatic arthritis and psoriasis following infliximab therapy. PloS One. 2014;9:e110657.

28. Hong K-M, Kim H-K, Park S-Y, Poojan S, Kim M-K, Sung J, et al. CD3Z hypermethylation is associated with severe clinical manifestations in systemic lupus erythematosus and reduces CD3ζ-chain expression in T cells. Rheumatol Oxf Engl. 2017;56:467–76.

29. Absher DM, Li X, Waite LL, Gibson A, Roberts K, Edberg J, et al. Genome-wide DNA methylation analysis of systemic lupus erythematosus reveals persistent hypomethylation of interferon genes and compositional changes to CD4+ T-cell populations. PLoS Genet. 2013;9:e1003678.

30. Linsley PS, Speake C, Whalen E, Chaussabel D. Copy number loss of the interferon gene cluster in melanomas is linked to reduced T cell infiltrate and poor patient prognosis. PloS One. 2014;9:e109760.

31. Ayano M, Tsukamoto H, Kohno K, Ueda N, Tanaka A, Mitoma H, et al. Increased CD226 Expression on CD8+ T Cells Is Associated with Upregulated Cytokine Production and Endothelial Cell Injury in Patients with Systemic Sclerosis. J Immunol Baltim Md 1950. 2015;195:892–900.

32. Walter GJ, Fleskens V, Frederiksen KS, Rajasekhar M, Menon B, Gerwien JG, et al. Phenotypic, Functional, and Gene Expression Profiling of Peripheral CD45RA+ and CD45RO+ CD4+CD25+CD127(low) Treg Cells in Patients With Chronic Rheumatoid Arthritis. Arthritis Rheumatol Hoboken NJ. 2016;68:103–16.

33. Banchereau R, Hong S, Cantarel B, Baldwin N, Baisch J, Edens M, et al. Personalized Immunomonitoring Uncovers Molecular Networks that Stratify Lupus Patients. Cell. 2016;165:551–65.

34. Hung T, Pratt GA, Sundararaman B, Townsend MJ, Chaivorapol C, Bhangale T, et al. The Ro60 autoantigen binds endogenous retroelements and regulates inflammatory gene expression. Science. 2015;350:455–9.

35. Hu S, Wang J, Meijer J, Ieong S, Xie Y, Yu T, et al. Salivary proteomic and genomic biomarkers for primary Sjögren's syndrome. Arthritis Rheum. 2007;56:3588–600.

36. Broeren MGA, de Vries M, Bennink MB, Arntz OJ, Blom AB, Koenders MI, et al. Disease-Regulated Gene Therapy with Anti-Inflammatory Interleukin-10 Under the Control of the CXCL10 Promoter for the Treatment of Rheumatoid Arthritis. Hum Gene Ther. 2016;27:244–54.

37. Rai R, Chauhan SK, Singh VV, Rai M, Rai G. RNA-seq Analysis Reveals Unique Transcriptome Signatures in Systemic Lupus Erythematosus Patients with Distinct Autoantibody Specificities. PloS One. 2016;11:e0166312.

38. Zhu H, Mi W, Luo H, Chen T, Liu S, Raman I, et al. Whole-genome transcription and DNA methylation analysis of peripheral blood mononuclear cells identified aberrant gene regulation pathways in systemic lupus erythematosus. Arthritis Res Ther. 2016;18:162.

39. Tasaki S, Suzuki K, Nishikawa A, Kassai Y, Takiguchi M, Kurisu R, et al. Multiomic disease signatures converge to cytotoxic CD8 T cells in primary Sjögren's syndrome. Ann Rheum Dis. 2017;76:1458–66.

40. Julià A, Absher D, López-Lasanta M, Palau N, Pluma A, Waite Jones L, et al. Epigenome-wide association study of rheumatoid arthritis identifies differentially methylated loci in B cells. Hum Mol Genet. 2017;26:2803–11.

41. Guo Y, Walsh AM, Fearon U, Smith MD, Wechalekar MD, Yin X, et al. CD40L-Dependent Pathway Is Active at Various Stages of Rheumatoid Arthritis Disease Progression. J Immunol Baltim Md 1950. 2017;198:4490–501.

42. Shchetynsky K, Diaz-Gallo L-M, Folkersen L, Hensvold AH, Catrina AI, Berg L, et al. Discovery of new candidate genes for rheumatoid arthritis through integration of genetic association data with expression pathway analysis. Arthritis Res Ther. 2017;19:19.
